# Patterns of genetic, phenotypic, and acoustic variation across a chiffchaff (*Phylloscopus collybita abietinus/tristis*) hybrid zone

**DOI:** 10.1002/ece3.2782

**Published:** 2017-03-02

**Authors:** Daria Shipilina, Maksym Serbyn, Vladimir Ivanitskii, Irina Marova, Niclas Backström

**Affiliations:** ^1^ Department of Vertebrate ZoologyLomonosov Moscow State UniversityMoscowRussia; ^2^ Department of PhysicsUniversity of CaliforniaBerkeleyCAUSA; ^3^ Department of Evolutionary BiologyEvolutionary Biology Centre (EBC)Uppsala UniversityUppsalaSweden

**Keywords:** Chiffchaff, genetic differentiation, hybrid zone, hybridization, introgression, speciation

## Abstract

Characterizing patterns of evolution of genetic and phenotypic divergence between incipient species is essential to understand how evolution of reproductive isolation proceeds. Hybrid zones are excellent for studying such processes, as they provide opportunities to assess trait variation in individuals with mixed genetic background and to quantify gene flow across different genomic regions. Here, we combine plumage, song, mtDNA and whole‐genome sequence data and analyze variation across a sympatric zone between the European and the Siberian chiffchaff (*Phylloscopus collybita abietinus/tristis*) to study how gene exchange between the lineages affects trait variation. Our results show that chiffchaff within the sympatric region show more extensive trait variation than allopatric birds, with a large proportion of individuals exhibiting intermediate phenotypic characters. The genomic differentiation between the subspecies is lower in sympatry than in allopatry and sympatric birds have a mix of genetic ancestry indicating extensive ongoing and past gene flow. Patterns of phenotypic and genetic variation also vary between regions within the hybrid zone, potentially reflecting differences in population densities, age of secondary contact, or differences in mate recognition or mate preference. The genomic data support the presence of two distinct genetic clades corresponding to allopatric *abietinus* and *tristis* and that genetic admixture is the force underlying trait variation in the sympatric region—the previously described subspecies (“*fulvescens*”) from the region is therefore not likely a distinct taxon. In addition, we conclude that subspecies identification based on appearance is uncertain as an individual with an apparently distinct phenotype can have a considerable proportion of the genome composed of mixed alleles, or even a major part of the genome introgressed from the other subspecies. Our results provide insights into the dynamics of admixture across subspecies boundaries and have implications for understanding speciation processes and for the identification of specific chiffchaff individuals based on phenotypic characters.

## 1 Introduction

Population divergence and speciation are the ultimate sources of biodiversity, and characterization of patterns and mechanisms during the speciation process is essential to understand how biodiversity is generated and maintained (Schluter, 2009). Speciation can be driven by different factors; a rather distinct partition is present between (1) speciation as a result of stochastic accumulation of mutations that contribute to reproductive isolation between geographically separated taxa and (2) speciation as a result of divergent natural selection, that is that local adaptation by itself drives the speciation process (Bierne, Gagnaire, & David, 2013; Coyne & Orr, 2004; Nosil & Feder, 2012; Nosil, Harmon, & Seehausen, 2009; Price, 2008; Schluter, 2009). Distinguishing between these scenarios makes it possible to understand the mechanisms underlying variation in observed patterns of biodiversity across regions. Hybrid zones, geographic regions where there is an overlap between distribution ranges and gene flow occurs between taxonomic units, can serve as “natural laboratories” allowing for close observation of microevolutionary processes that may lead to population differentiation and, ultimately, speciation (Abbott, Barton, & Good, 2016; Barton & Hewitt, 1989; Payseur & Rieseberg, 2016). In the Palearctic and Nearctic regions, such hybrid zones are naturally most often secondary contact zones, the results of distribution range expansions following the latest glaciation period (Hewitt, 2004, 2011). By studying the secondary contact hybrid zones closely, we may investigate if there are any barriers to gene flow between the taxa involved and combine that with information about potential forces behind divergence, that is if taxa have diverged because of random acquisition of incompatibility alleles (observed trait differences not related to ecology) or if adaptation has played a major role in the divergence process (potential trait differences clearly associated with ecology) (Toews & Irwin, 2008). In addition, studying secondary contact zones can give information about the designation of lineages with unclear taxonomic status by allowing for quantification of genetic differentiation and potential introgression between involved taxa (Abbott et al., 2016; Delmore et al., 2015; Payseur & Rieseberg, 2016).

The Old World leaf warbler group (genus *Phylloscopus*) contains a large set of species that express considerable resemblance in plumage coloration and morphology (del Hoyo, Elliot, & Christie, 2006). The large number of species within the genus in combination with a substantial variation in the degree of phenotypic differentiation between species pairs has helped provide understanding of what types of premating reproductive isolation barriers act at different stages of the speciation succession (Bensch, Grahn, Müller, Gay, & Åkesson, 2009; Price, 2010). For instance, pioneering work focusing on genus level phylogenetic analyses (Bensch, Irwin, Irwin, Kvist, & Åkesson, 2006; Helbig et al., 1996), targeted analysis of the genetic underpinnings of migratory behavior (Bensch, Åkesson, & Irwin, 2002; Bensch et al., 2009; Lundberg, Åkesson, & Bensch, 2011; Lundberg et al., 2013), the role of sex‐chromosomes in driving divergence (Helbig, Salomon, Bensch, & Seibold, 2001) and genomic differentiation between populations of a “ring species” (Alcaide, Scordato, Price, & Irwin, 2014; Irwin, Alcaide, Delmore, Irwin, & Owens, 2016) has increased the understanding of the forces underlying reproductive isolation between some taxa within the genus, but we are still far from a detailed understanding of the mechanisms underlying the divergence processes.

The chiffchaff superspecies complex contains four distinct species and a set of subspecies widely distributed within the Palearctic region (Figure S1). The Canarian chiffchaff (*P. canariensis*), including subspecies *canariensis* and, as of now extinct, *exsul*, inhabits the Canary Islands. The distribution range of the Iberian chiffchaff (*P. ibericus*) spans the Iberian Peninsula and the Pyrenees and southwestern France. The mountain chiffchaff (*P. sindianus*) breeds in high‐altitude habitats in the Caucasus Mountains (*P. s. lorenzii*) and in parts of eastern–central Asia (*P. s. sindianus*). The common chiffchaff (*P. collybita*) has traditionally been separated into six different subspecies: *collybita* (distributed throughout southern and central Europe), *brevirostris* (northern Asia Minor), *caucasicus* (lowlands in Caucasus), *menzbieri* (Kopet Dagh and southwestern Transcaspia), *abietinus* (north‐eastern Europe to the Ural Mountains), and *tristis* (Ural Mountains to east Siberia; Stepanyan, 1990; Svensson, 1992). However, the taxonomic status of *tristis* has been a topic of some debate over whether or not it should be designated full species status (del Hoyo et al., 2006). In this study, we will refer to both *abietinus* and *tristis* as subspecies of the common chiffchaff (*P. collybita*).

In some areas of the superspecies distribution range, chiffchaff species and/or subspecies occur in sympatry. The largest such zone was identified already in the late nineteenth century (Sushkin, 1897). This region is likely a secondary contact zone formed during the recolonization from glacial refugia following the retraction of the ice cover after the latest glaciation period, but it has still not been described in much detail. The zone more or less coincides with the Ural Mountains all the way from the southern edge to the White Sea (Komarova & Shipilina, 2010; Marova, Fedorov, Shipilina, & Alekseev, 2009) and includes two subspecies of the common chiffchaff: the “Siberian” chiffchaff (*P. c. tristis*) and the “European” common chiffchaff (*P. c. abietinus*) – two subspecies with differences in habitat preference, body size, plumage characteristics, song type, and migration routes/wintering quarters (Dean & Svensson, 2005; Helbig et al., 1996; Marova & Alekseev, 2008; Ticehurst, 1938). The interindividual variation in plumage coloration, body size, and song type is more pronounced within the sympatric region than within allopatric populations of either *abietinus* or *tristis*. In particular, some individuals performing intermediate song, so‐called mixed singers (Lindholm, 2008; Marova & Leonovich, 1993) and intermediate plumage phenotypes have previously been identified in the sympatric zone (Marova et al., 2009). This particular region has also been thought to be inhabited by a third subspecies, “*P. c. fulvescens*”, expressing intermediate characters (plumage phenotype and song type) to those observed in *abietinus* and *tristis* (Dean & Svensson, 2005; Severtsov, 1873; Stepanyan, 1990). However, a set of recent studies are not consistent with this hypothesis: a weak association between song type, plumage color, and mitochondrial haplotype has been found within the sympatric region but a considerable proportion of birds deviated from the pattern (Marova & Alekseev, 2008; Marova, Shipilina, Fedorov, & Ivanitskii, 2013; Marova et al., 2009). This is in agreement with recurrent intercrossing between subspecies in the region. In further support of that, a small‐scale genetic analysis using restriction enzyme digestion of a single mitochondrial gene showed haplotype differentiation between *abietinus* and *tristis* (Helbig et al., 1996; Marova et al., 2009), but birds with distinct *abietinus* phenotype sometimes carried the *tristis* mitochondrial haplotype or had the typical *tristis* song dialect and vice versa (Marova et al., 2009, 2013). However, information from a single, maternally inherited genetic marker does not necessarily provide conclusive evidence for genetic admixture and rule out the presence of a third subspecies. To make a more detailed investigation of the genomic consequences of admixture between *abietinus* and *tristis* in regions of sympatry and to ultimately test the validity of the previously described “*fulvencens*” subspecies, we combine previously available data on morphology, acoustics, and mitochondrial DNA (Marova et al., 2009, 2013) with novel whole‐genome sequence data. We hypothesize that reproductive barriers between *abietinus* and *tristis* are incomplete and that genetic admixture has given rise to the intermediate phenotypes frequently observed within the sympatric zone. In addition, to test if relative population density and time since recolonization may affect patterns of genetic variation in intermixed populations, we analyze samples from two distinct geographic locations that differ in relative densities of *abietinus* and *tristis* and in distance to potential glacial refugia: the southernmost (southern Ural mountains, predominantly mixed forest, closer to glacial refuge) and northernmost (Arkhangelsk region, boreal forest, further away from glacial refuge) parts of the sympatric zone.

## 2 Methods

### 2.1 Capture of specimens and DNA extractions

Active chiffchaff males were captured by playback and mist netting during breeding seasons of 2007–2009 (see Figure S1 for sampling locations and sample sizes). The data were collected in the following way: first, male song was recorded for at least three minutes. Then, individuals were caught in a mist net by sound trapping. We quantified plumage coloration (morphotype) and took a blood sample. The key for the morphological identification of the subspecies is yellow (lipochrome) coloration intensity on the ventral side of the body: throat, breast, and belly (Svensson, 1992; Ticehurst, 1938). Individuals were classified as *abitinus* if they had bright yellow stripes on the breast and yellow and green tones in overall coloration and as *tristis* if they showed absence of any yellow hues in coloration except on the underwing (Svensson, 1992; Ticehurst, 1938). Birds classified as intermediate had yellowish feathers to varying extents, but always clearly less than *abietinus* or more than *tristis* (Marova et al., 2009). In total, 245 individuals were included in this study; however, due to specifics of the sampling process, we were not able to characterize all of them for all sets of parameters: morphotype, acoustics, mtDNA haplotype, and nuclear SNPs. Of the 245 sampled individuals, 228 birds were characterized as *abietinus*,*tristis,* or intermediate based on morphological characters (Marova & Alekseev, 2008; Marova et al., 2009).

For 138 birds, we could record the song and make high‐quality sonograms for quantification of acoustic patterns (Marova & Alekseev, 2008; Marova et al., 2009). Songs of *tristis* and *abietinus* can be easily distinguished by ear or by sonogram analysis due to significant differences in tempo and structure (Ticehurst, 1938; Marova et al., 2009). A clear distinction between song types is the presence of ascending notes in the typical *tristis* song and the absence of such notes in the song of *abietinus,* the considerably shorter interval between notes in *tristis* (4.7–7.2 notes per second) than in *abietinus* (2.8–3.3 notes per second), and the almost nonoverlapping frequency ranges of notes (*abietinus*: 3.7–4.6 kHz; *tristis*: 2.9–3.7 kHz). To designate recorded birds to a specific song type, we calculated the percentage of ascending elements in three randomly selected three‐second long intervals of songs of each recorded male (visual analysis of spectrograms) and measured the frequency range and note rate using Syrinx 2.3 (J. Burt, Seattle, WA, USA).

Blood samples were collected from specimens by careful piercing of the brachial vein using standard syringes. In total, blood samples from 197 of the 245 chiffchaffs were collected and stored on filter paper. Sampling sites were distributed both within the allopatric ranges of *abietinus* (*n* = 16) and *tristis* (*n* = 37) and within both the northern (in total 67 samples; based on plumage characters, these were 24 *tristis*, 18 *abietinus,* and 21 with intermediate plumage phenotype, for 4 birds morphology was not determined) and the southern (in total 125 samples; 41 *tristis*, 44 *abietinus,* and 27 with intermediate phenotype, for 13 birds phenotype was not determined) part of the sympatric zone (Figure S1). All samples from the northern sympatric zone are novel to this study while the majority of the data from allopatric regions and the southern sympatric zone have been used in two previous studies of plumage, song, and mtDNA variation (Marova et al., 2009, 2013). In total, there were 105 individuals that were analyzed using all three characteristics: morphotype, song type, and mtDNA haplotype. DNA from whole blood samples was extracted using a standard phenol–chloroform protocol (Sambrook, Fritsch, & Maniatis, 1989). The quantity and quality of the DNA preparations was assessed with agarose gel (1%) electrophoresis and measurement with the Qubit (Thermo Fisher Scientific Inc.) and the NanoDrop (Thermo Fisher Scientific Inc.) instruments.

### 2.2 mtDNA analyses

One hundred and ninety‐two birds could be scored for mtDNA haplotype at a region in the Cytochrome B (*CytB*) locus by restriction analysis. We analyzed the same 389‐bp‐long region that has previously been published for the chiffchaff (GenBank accession entries: Z73479.1, Z73482.1). Within this region of *CytB,* five SNPs have previously been found to be diagnostic between allopatric European and Siberian chiffchaff (Helbig et al., 1996). For restriction analysis, we chose two of those diagnostic SNPs which allowed us to distinguish between distinct allopatric haplotypes and a third SNP that made it possible to identify the presence of a previously identified haplotype that also has been found to be unique to *tristis* and that differs to the most common *tristis* haplotype at a single nucleotide position (Marova et al., 2009). Restriction analysis was performed using the endonuclease Hinf I (GANTC). For each sample, we used 0.3 μl of Hinf1 mixed with Buffer R (1 μl) and bi‐distilled water (4 μl), and 5 μl of extracted DNA was added to the mix. The restriction reagent mix was held at 37°C for 12 hr, and fragment types were scored by regular polyacrylamide gel electrophoresis. All scored mtDNA variants have been submitted to datadryad.org and are available under doi: 10.5061/dryad.g74v0.

### 2.3 Whole‐genome sequencing and SNP detection

Of the 223 sampled specimens that were scored for plumage color, we selected 10 individuals with the highest DNA yield from each subspecies and each area (i.e., 10 allopatric *tristis*, 10 allopatric *abietinus*, 10 sympatric *tristis*, and 10 sympatric *abietinus*) and prepared standard insert size (380 bp) libraries for 100 bp paired‐end sequencing on the Illumina HiSeq2000 platform. Library preparation and sequencing was performed by the SNP&SEQ Technology Platform in Uppsala. The sequencing yield was in total 147 Gb and varied between 2.7 and 5.1 Gb for each sample. The range for individual samples was 1.6–4.3 Gb after filtering out bases with *Q*‐scores <30 using ConDeTri (Smeds & Künstner, 2011), in total 113 Gb. The quality‐filtered nuclear sequence reads were mapped to the *Ficedula albicollis* genome sequence (Ellegren et al., 2012; Kawakami et al., 2014) using the Burrows–Wheeler algorithm as implemented in the software BWA (version 0.5.9, Li & Durbin, 2010). SAMtools (Li et al., 2009) was used to convert files to BAM format and to visually inspect some of the alignments. We subsequently merged all mapped reads from individual samples into a single BAM file using Picard Tools (http://broadinstitute.github.io/picard/). We applied the methods in the package GATK (McKenna et al., 2010) for nucleotide quality score recalibration, local insertion/deletion realignment and removal of duplicates, and called SNPs across all samples using filtering parameters and SNP quality recalibration recommended in the GATK Best Practices (De Pristo et al., 2011; Van der Auwera et al., 2013), without a training set as no genomic SNP data were previously available for these subspecies. Specifically, this was performed in two steps: first, the RealignerTargetCreator and IndelRealigner packages were used for local realignment to purge reads that might have been erroneously mapped, and then, the UnifiedGenotyper package was used to call high‐quality SNPs (criteria: *Q*‐score >30, minimum coverage 5 reads per SNP per individual) and save these in vcf format files. The SNP calling procedure resulted in 4.9 million high‐quality SNPs that could be used for downstream analysis (Figure S2).

### 2.4 Analyses of genomic SNP variation

Processing of vcf files was performed in the package PyVCF in the Python environment (https://github.com/jamescasbon/PyVCF). Two further filtering steps were performed to generate the final data sets used for analysis. We initially applied stringent criteria and selected a set of SNPs, in which we had high‐quality SNP score information from at least 9 of the 10 individuals from each population, respectively. After this filtering step, only 18,014 SNPs were retained—this is from here on referred to as the “high‐stringency data set”. This high‐stringency data set was subsequently used to quantify and visualize patterns of differentiation between populations but did not contain more than 50 fixed differences (50 fixed differences/18,014 SNPs in total = 0.3%) between *abietinus* and *tristis*. To obtain a larger set of polymorphisms for assessment of rates of fixed, private, and shared polymorphisms between and within *abietinus* and *tristis*, respectively, and for analysis of genomic composition of birds sampled in the sympatric region, we selected sites that were present in at least six different individuals in both allopatric *abietinus* and allopatric *tristis*. After that filtering step, 1,233,236 high‐quality SNPs were retained—from here on referred to the “low‐stringency data set”. It should be noted that the relative frequency of fixed differences did not change for the lower stringency data set (3,555 fixed SNPs/1,233,236 SNPs in total = 0.3%). All genotypes have been submitted to datadryad.org and are available under doi: 10.5061/dryad.g74v0.

We used in‐house developed Python scripts (available upon request) to calculate the number of fixed substitutions between allopatric populations, as well as private and shared polymorphisms within populations using the larger set of SNPs (1,233,236 SNPs). Fixation indices (*F*_ST_) were calculated for both allopatric and sympatric population comparisons (allopatric *abietinus* versus allopatric *tristis* and sympatric *abietinus* versus sympatric *tristis*) using the high‐stringency data set (18,014 SNPs) and the package PopGenome (http://popgenome.weebly.com) in the R environment (Pfeifer, Wittelsbuerger, Ramos‐Onsins, & Lercher, 2014). Error estimates were generated by jackknife resampling over sets of 50 SNPs. In addition to overall *F*_ST_‐estimates, the degree of genetic differentiation between all 40 samples that were whole‐genome sequenced grouped as (1) allopatric *abietinus*, (2) allopatric *tristis*, (3) southern sympatric samples (sample id:s S1‐S10), and (4) northern sympatric samples (sample id:s N1‐N10), respectively) was visualized by using a principal component analysis of genetic variation as implemented in the package smartPCA (EigenSoft) for R (Patterson, Price, & Reich, 2006). Preparation of data files for that analysis was carried out using the program PyVCF (https://github.com/jamescasbon/PyVCF), and graphs were constructed using the ggplot2 library in R. In addition, we applied a population assignment analysis based on allele frequencies across the high‐stringency SNPs as implemented in the program STRUCTURE 2.3.4 (Pritchard, Stephens, & Donnelly, 2000) using the admixture model. We ran Structure for 50,000 iterations after a burn‐in of 20,000 iterations, five times for each of seven *K* values (*K* = 1 to *K* = 7 populations) and evaluated the optimal *K* as described in Evanno et al. (2005). All five independent runs for each *K* resulted in consistent results. A summary of all data treatments and analytical steps for each data set is presented in Figure S2.

## 3 Results

Within the allopatric regions, all birds were scored as typical *abietinus* or *tristis*, respectively, both regarding plumage characteristics and song type. In the sympatric regions, the variation in plumage and song type was greater with several individuals scored as intermediate/mixed phenotypes (summarized in Table 1). Specifically, we found that 33% of all sampled individuals had intermediate plumage characters (only pale yellow hue and negligible amount of yellow feathers on ventral side) in the northern sympatric region, and the corresponding value for the southern sympatric region was 24%. For the distribution of song types, there was again a considerable proportion of individuals in the sympatric regions performing mixed song—that is a mixture of typical *tristis* and *abietinus* notes and intermediate frequency range and song speed (notes per time unit)—63% in the northern and 40% in the southern sympatric region.

**Table 1:** Summary of morphotype, song type, and genetic characteristics of chiffchaff sampled within the sympatric zone (northern and southern part and in total)

	Northern part (%)	Southern part (%)	Total (%)
Morphotype (*n* = 175)			
*Abietinus*	18 (28.6)	44 (39.3)	62 (35.4)
*Tristis*	24 (38.1)	41 (36.6)	65 (37.1)
Intermediate	21 (33.3)	27 (24.1)	48 (27.4)
Song type (*n* = 80)			
*Abietinus*	3 (11.1)	10 (18.9)	13 (16.3)
*Tristis*	7 (25.9)	22 (41.5)	29 (36.3)
Mixed	17 (63.0)	21 (39.6)	38 (47.5)
mtDNA (*n* = 144)			
*Abietinus*	3 (5.7)	20 (22.0)	23 (16.0)
*Tristis*	50 (94.3)	71 (78.0)	121 (84.0)
Nuclear SNPs (*n* = 20)			
Diagnostic *abietinus*, %	17.4	34.8	26.1
Diagnostic *tristis*, %	68.2	59.5	63.9
Heterozygous sites, %	14.4	5.7	10.1

Numbers are counts (percentages of total in brackets) of individuals within each respective class of characters. For the nuclear SNPs (total *n* = 1,233,236 SNPs, low‐stringency data set), the average proportions of diagnostic and heterozygous SNPs across individuals are given for each respective sympatric region and in total.

**Figure 1:**
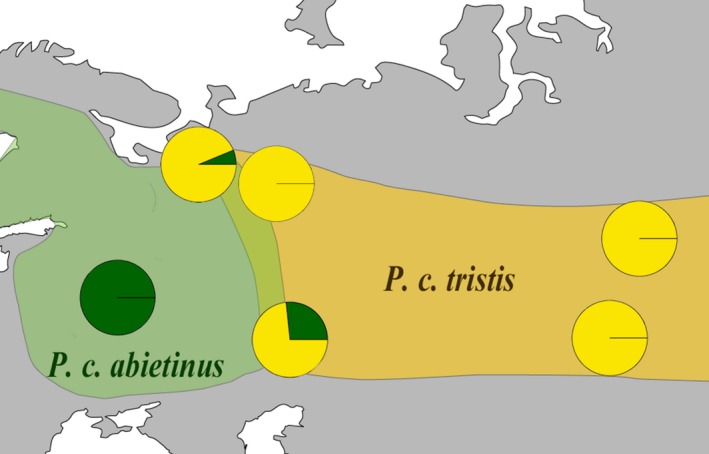
Distribution of scored mitochondrial haplotypes across the European and Siberian chiffchaff ranges with particular focus on the sympatric zone. The two identified *tristis* haplotypes that only differ at a single nucleotide position have been grouped and are presented in yellow, and the diagnostic *abietinus* haplotype is given in green

We obtained high‐quality data for the mitochondrial *CytB* gene for three positions (SNPs) using restriction analysis in 193 individuals of allopatric *abietinus* (*n* = 12) and *tristis* (*n* = 37) and birds from both the northern (*n* = 53) and the southern part (*n* = 91) of the sympatric zone. Allopatric birds all had distinct mtDNA haplotypes (a single haplotype in Europe and a single haplotype in Siberia). Both in the northern and in the southern sympatric regions, we observed a mixture of these haplotypes, along with a previously identified third haplotype (*tristis‐2*, haplotype found in 21 birds) that differs from the diagnostic *tristis* haplotype found in allopatry at a single nucleotide position (Marova et al., 2009). Distribution of haplotypes within the geographic regions are shown in Figure 1 where the two similar *tristis* haplotypes are merged in a single group.

From our data and previous analyses of morphological and acoustic characteristics (Marova & Alekseev, 2008; Marova et al., 2009), we know that the southern Urals (southern sympatric zone) is a clear transition zone of morphotypes and dialects. We checked if this pattern fitted our distribution of mitochondrial haplotypes and the trend was apparent with a higher frequency of diagnostic European haplotypes in the western part (65%; *n* = 13/20) than in the eastern part (11%; *n* = 6/55) (χ^2^ = 19.9, *df* = 1, *p*‐value = 8.1 × 10^−6^). In the northern sympatric region, only three of the 53 males had the *abietinus* mtDNA haplotype, and a quantification of frequency differences across the zone had no power. A detailed summary of the counts of mitochondrial haplotypes, morphotypes, and dialects in the sympatric region is presented in Table 1.

Our combination of quantification of song and morphology together with mtDNA haplotyping and characterization of nuclear SNPs allow us to characterize each male chiffchaff in the sample from the sympatric zone using a combination of characteristics: morphotype (plumage characteristics), acoustics/song type, and genetic data (mtDNA haplotype, nuclear SNP alleles). In summary, we find a pronounced mosaic in the distribution of characteristics (Table 1). Some examples to point out relate to the difference between appearance and genomic composition. First, a large proportion of individuals with distinct *abietinus* morphotype had *tristis* mtDNA (58%), while the opposite was found to be rare (1 sample, 1.8%). Second, all birds except one (97.5%) with intermediate morphotype had *tristis* mtDNA type. Third, 15 of 43 males with the *tristis* mtDNA sang the distinct *tristis* song while three males performed pure *abietinus* song, and as many as 25 were scored as mixed singers. Of 10 males with *abietinus* mtDNA and morphotype, three were scored as mixed singers, seven were singing pure *abietinus* song type, and none was found to perform *tristis* song type. Thus, of 53 males with known mtDNA type and scored dialect, as many as 28 (53%) performed mixed song and three males (5.6%) performed typical *abietinus* song, despite having *tristis* mtDNA.

**Figure 2:**
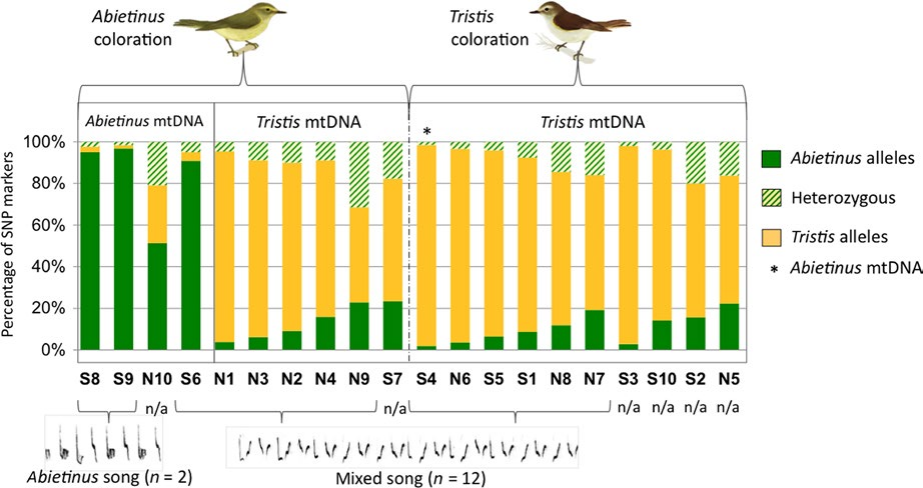
The proportion of diagnostic SNPs in the 20 samples from the sympatric zone that were selected for whole‐genome sequencing. The bars indicate the proportions that are of either *abietinus* (green) or *tristis* (yellow) origin. The fraction of heterozygous SNPs in an individual is marked with barred green. Samples are grouped by morphotype, mtDNA haplotype, and song type. Individuals S1–S10 are samples from the southern sympatric region, and individuals N1–N10 are samples from the northern sympatric region

To compare the genomic composition of whole‐genome sequenced samples from within the sympatric zone with their phenotypes, we assessed the presence of SNPs found to be fixed (low‐stringency data set, *n* = 3,555) between allopatric *abietinus* and *tristis*. This is summarized in Figure 2. For birds with the *tristis* morphotype, a majority of the nuclear SNPs were homozygous for diagnostic *tristis* alleles, while there was much more variation for birds with the *abietinus* morphotype, and there was a strong association between the proportion of diagnostic SNPs and the mtDNA haplotype. However, morphotype, mtDNA type, and genomic composition did not always match. For example, sample N1 had typical *abietinus* morphotype, but a very large fraction of *tristis* diagnostic SNPs and a *tristis* mtDNA haplotype, and sample S4 had typical *tristis* morphotype and was homozygous for >95% of diagnostic *tristis* alleles but actually had *abietinus* mtDNA (Figure 2). Among the samples from the sympatric zone that were scored for genome‐wide SNP polymorphisms, we had acoustic information for 14. Two performed typical *abietinus* song and both of these had a large fraction of *abietinus* diagnostic genetic markers (>95%) and *abietinus* mtDNA type. Of the 12 individuals with mixed song type, six had *tristis* morphotype and six had *abietinus* morphotype. A majority of these individuals (10/12) had a combination with a high fraction of *tristis* diagnostic SNPs and *tristis* mtDNA, while one bird (S6) had *abietinus* mtDNA and a large proportion of *abietinus* diagnostic SNPs. Another bird (S4 again) had *abietinus* mtDNA but >95% of diagnostic *tristis* nuclear SNP alleles (Figure 2).

**Table 2:** Summary of proportions (in %) of fixed, shared, and private polymorphisms in the European (*abietinus*) and the Siberian chiffchaff (*tristis*) from zones of allopatry and sympatry, respectively (total *n* = 1,233,236 SNPs, low‐stringency data set)

	Fixed	Shared	Private (*abietinus*)	Private (*tristis*)
Allopatry	0.3	48.2	22.3	29.1
Sympatry	0.03	60.9	17.5	21.6

To quantify the proportion of shared genetic variation in allopatric and sympatric regions, we calculated the percentage of fixed, private, and shared polymorphisms (low‐stringency data set) between both allopatric and sympatric populations. For samples from the sympatric region, we grouped the data based on morphotype, regardless of from what part of the area in the sympatric zone the sample originated. We found that the percentage of fixed differences between representatives of the *abietinus* and *tristis* morphotypes was lower between sympatric (0.03%) than between allopatric populations (0.3%), and the proportion of shared polymorphisms was higher between sympatric (60.9%) than between allopatric populations (48.2%) (test of proportions, χ^2^ = 3452, *df* = 1, *p*‐value <2.2 × 10^−16^; Table 2). A further assessment of the level of genetic differentiation between *abietinus* and *tristis* was performed by estimating the fixation index (*F*_ST_) for all nuclear SNPs. A summary of these estimates is presented in Table 3. The level of differentiation was higher between allopatric (*F*_ST_ = 0.062) than between sympatric (*F*_ST_ = 0.006) *abietinus* and *tristis*. We also compared populations sampled in different parts within the sympatric zone. We observed a slightly lower level of genetic differentiation between sympatric *abietinus* and *tristis* populations within the southern region (*F*_ST_ = 0.013) than within the northern region (*F*_ST_ = 0.016).

**Table 3:** Estimates of the mean fixation indices (*F*_ST_) between different European (*abietinus*) and Siberian (*tristis*) chiffchaff populations for nuclear SNPs (high‐stringency data set)

Populations compared	*F*_ST_
Allopatric *tristis* vs allopatric *abietinus*	0.062 ± 0.003
Sympatric *tristis* vs sympatric *abietinus*	0.006 ± 0.0008
N. sympatric zone *tristis* vs *abietinus*	0.013 ± 0.0009
S. sympatric zone *tristis* vs *abietinus*	0.016 ± 0.0011

**Figure 3:**
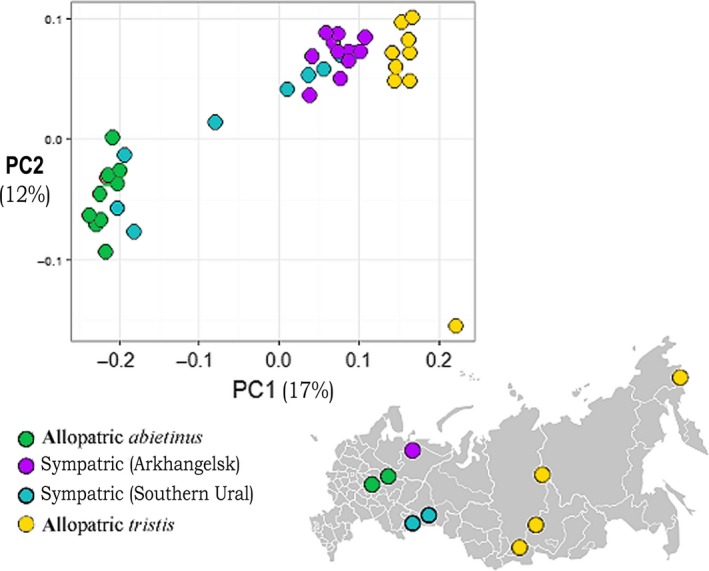
Principal component analysis (PCA) illustrating the genetic differentiation across samples from both the allopatric (green = *abietinus* (A1–A10), yellow = *tristis* (T1–T10)), and the sympatric (purple = samples from N. sympatric zone (N1–N10), blue = samples from S. sympatric zone (S1–S10)) regions (*n* = 18,014 SNPs). The map illustrates the geographic locations of samples from each respective group

**Figure 4:**
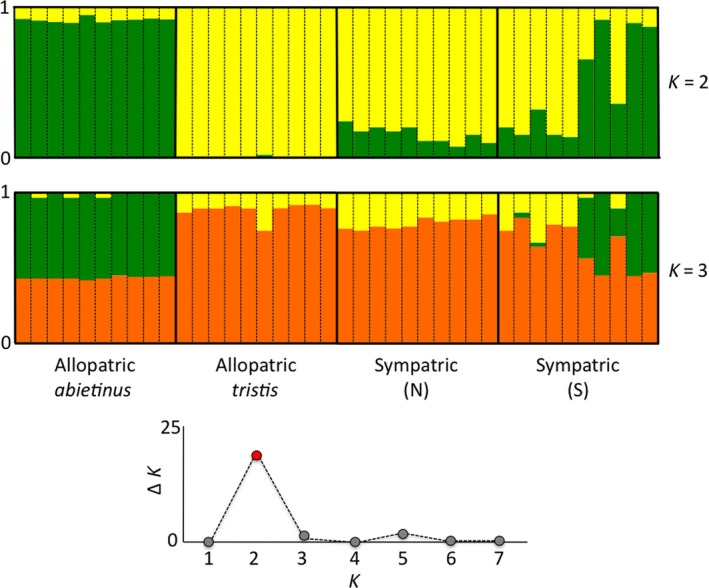
Illustration of the STRUCTURE analysis using the high‐stringency data set (*n* = 18,014 SNPs) for *K* = 2 (top panel) and *K* = 3 (bottom panel) clusters. The allopatric samples are represented in Sections 1 (*abietinus*: A1–A10) and 2 (*tristis*: T1–T10), and samples from the sympatric region are presented in sections 3 (North: N1–N10) and 4 (South: S1–S10). The graph at the bottom shows the evaluation of optimal *K* from *K* = 1 to *K* = 7 as described in Evanno et al. (2005)

Visualization/quantification of genetic differentiation across samples was performed using both Principal component analysis (PCA) and clustering analysis (STRUCTURE) of the high‐stringency SNP data. Figure 3 illustrates the outcome of the PCA analysis using the first and second principal components (PC1 and PC2 explain 17% and 12% of the total variation, respectively). Allopatric *abietinus* and *tristis* were well separated from each other, especially along the axis representing principal component 1 and both clustered together in dense groups with the exception of one *tristis* sample that showed considerable differentiation from other *tristis* samples, mainly along the PC2 axis. Samples from the sympatric regions clearly occupy an intermediate position between the allopatric individuals, the samples from the northern sympatric zone rather close to the allopatric *tristis* samples, while the samples from the southern sympatric zone are more scattered with some closer clustering together with allopatric *abietinus* samples (Figure 3). The STRUCTURE (Pritchard et al., 2000) analysis clearly separated allopatric *abietinus* and *tristis* and again, samples from the sympatric zone showed varying degrees of allele sharing to either subspecies indicating that the samples from the sympatric zone have mixed ancestry between *abietinus* and *tristis* (Figure 4, *K* = 2). To test the hypothesis of presence of a third subspecies (*fulvescens*), we modeled the presence of different numbers of discrete clades within the investigated region (*K* = 1 to *K *= 7) and evaluated the number of clades with highest likelihood (Evanno et al. 2005). The results showed that *K* = 2 had the highest likelihood (Figure 4, detailed results for *K* = 2 and *K* = 3 are given), and there was no support for the presence of a distinct clade that differs from *abietinus* and *tristis,* again supporting the hypothesis that birds in the sympatric region have a genomic composition with admixed *abietinus* and *tristis* alleles (Figure 4).

## 4 Discussion

In this study, a large number of male chiffchaff were sampled across the distribution ranges of subspecies *abietinus* and *tristis*. By combining data on morphology and acoustics with mtDNA haplotype information and a set of genome‐wide SNPs, we analyzed patterns of phenotypic and genetic diversity and differentiation within allopatric and sympatric regions. Below we discuss these patterns of variation in light of species history and identification issues.

In allopatric regions, we observed limited variation in morphotype and song type across individuals within *abietinus* and within *tristis* and each subspecies was carrying a distinct mtDNA haplotype. In the sympatric region, however, sampled individuals showed considerable variation in both traits and we found a mixture of the two distinct mtDNA haplotypes (together with 21 cases where birds carried a third mtDNA haplotype that previously has been shown to differ from the typical *tristis* haplotype at a single position). This supports previous studies indicating presence of mtDNA introgression between the two subspecies (Marova et al., 2009, 2013) but does not provide any information about potential nuclear genomic gene exchange. To investigate the patterns of admixture in more detail, we analyzed genome‐wide genetic variation using SNP data. We conducted a set of different types of analyses using the nuclear SNPs that all support considerable genetic admixture: principal component analysis, assignment tests, assessment of genetic differentiation, and analysis of the contribution of subspecies specific “diagnostic” genetic markers to samples within the zone of sympatry. The principal component analysis showed a clear separation of the allopatric populations of *abietinus* and *tristis* into two distinct clusters and an intermediate position for most of the investigated samples from within the sympatric zone. This indicates that gene flow between the two subspecies has resulted in higher degree of allele sharing between individuals in sympatry. Support for this also comes from our clustering analysis which has the highest likelihood for two distinct populations (allopatric *abietinus* and *tristis*, respectively), while samples from the sympatric zone have a genetic set‐up built up by intermixed contributions from the two subspecies. When assessing the fractions of shared and fixed polymorphisms and estimating the global genetic differentiation (*F*_ST_), we observed a substantially lower level of fixed differences (higher genetic differentiation) and higher fraction of shared polymorphisms (lower genetic differentiation) between subspecies in sympatry than in allopatry, again supporting a scenario with admixture and allele sharing in the region where subspecies ranges overlap, although this should be taken as circumstantial rather than hard evidence, as the grouping of individuals in the sympatric region was based on morphotype alone.

It should be noted that the PCA analysis revealed that one *tristis* sample showed clear differentiation to all other *tristis* samples. This sample originates from the Chukotka region in far eastern Siberia and is hence considerably geographically separated from all other *tristis*. In addition, the *tristis‐2* mtDNA haplotype was not found in allopatric *tristis*. These observations indicate that there is genetic structure also within the *tristis* subspecies, but extended sampling is obviously necessary to quantify the degree of potential differentiation between *tristis* populations and how that relates to observed “intrasubspecific” variation in phenotypic traits such as song and plumage color (Marova et al., 2009, 2013). We also observed a somewhat higher fraction of private polymorphisms in *tristis* (29% of all SNPs) than in *abietinus* (22% of all SNPs). That indicates a slightly larger effective population size in *tristis* than in *abietinus* and is in agreement with the larger distribution range but could also be a consequence of that current *tristis* populations expanded from multiple glacial refugia (Nazarenko, 1982, 1985; Vorontsov, 1949).

Within the sympatric region, we found strong association between genetic set‐up and appearance in some individuals, two birds with *abietinus* morphotype and song also had the *abietinus* mtDNA and close to 100% *abietinus* diagnostic nuclear SNPs, and 9 birds with *tristis* morphotype had *tristis* mtDNA and a major fraction of *tristis* diagnostic nuclear SNPs. (It should be noted that we also observe a small fraction of heterozygous sites and sites diagnostic for the other subspecies (i.e., *abietinus* alleles in a *tristis* sample) in these particular individuals. It could either indicate that i) these samples are representatives for multi‐generation recurrent backcrossings into one of the parental subspecies, and/or ii) that our selection of diagnostic SNPs contains a minor proportion of markers that in fact are shared or private). However, six birds that were scored as *tristis* based on plumage characters and mtDNA performed the mixed song type, and there were also several examples where the appearance and song did not match the genetic set‐up. The most evident cases include five birds with typical *abietinus* morphotype that actually had the *tristis* mtDNA, and a majority of diagnostic *tristis* nuclear SNPs that also performed mixed song and one bird, also a mixed singer, that had the typical *tristis* morphotype and almost 100% *tristis* diagnostic nuclear SNPs but *abietinus* mtDNA. This is in line with previous analysis of correlations between mtDNA and phenotype in chiffchaff sampled across ringing stations in Netherlands where a large proportion of birds identified as *abietinus* in hand actually had a typical *tristis* mtDNA type (De Knijff, van der Spek, & Fischer, 2012) and indicates that species determination based on morphology can be uncertain. Hence, many birds that appear as obvious, distinct *abietinus* or *tristis* can harbor a genetic set‐up that is a mix between species or even almost completely fixed for foreign alleles. We conclude from the combined analyses of phenotypes, mtDNA, and genome‐wide SNPs in both allopatry and sympatry that extensive historical and ongoing gene flow between the subspecies have resulted in a transition zone of morphotypes and song types.

On a related note, it might be of interest to use our results to speculate in more detail around the taxonomic status of chiffchaff populations, inhabiting the 1,500 km long region from the Arkhangelsk region in the north to the southern Ural Mountains in the south and also the region covering parts of the west Siberian plain. Based on spearheading naturalist work during the nineteenth century (Severtsov, 1873), it has been suggested that the region harbors a distinct chiffchaff taxon, “*fulvescens*”, described as expressing intermediate characters (morphotype, song) compared to *abietinus* and *tristis* (Dean & Svensson, 2005). Later observations suggested that chiffchaff sampled in the west Siberian plain area (between the Ural mountains and the Yenisei river) sometimes showed a morphotype that deviated slightly from *tristis* samples from east of the Yenisei river, in essence showing partly more yellowish plumage (Dean & Svensson, 2005). Additional studies in the southern Urals concluded that birds in this region showing intermediate morphotype and mixed song probably were hybrids and/or backcrosses between *abietinus* and *tristis* (Marova et al., 2009, 2013; Ticehurst, 1938), initially these forms were named “*riphaeus”* (Ticehurst, 1938). As mentioned above, we observed a clear transition zone between distinct *abietinus* and *tristis* across this region, the diagnostic mtDNA haplotypes were intermixed in the area and genome‐wide nuclear SNP variation did not indicate any additional structuring besides that allopatric *abietinus* and *tristis* are genetically distinct, but exchange substantial portions of the genome where distribution ranges overlap. This leads to the conclusion that the “*fulvescens”* (“*riphaeus*”) form represents individuals with a mixed genetic set‐up from both *tristis* and *abietinus* rather than a distinct taxon and that the most plausible explanation for the occurrence of “*fulvescens”* type birds east of the immediate contact zone is that these represent individuals with introgressed *abietinus* alleles on a *tristis* genomic background (see also discussion on directional gene flow below). This scenario could easily be envisioned if hybrids and backcrosses preferentially interbreed with “pure” *tristis* generating a more diluted transition zone east of the immediate contact zone.

The evolution of reproductive isolation is generally a long‐drawn‐out process, initiated by divergence in traits related to prezygotic barriers and ending with postzygotic genetic incompatibilities (Coyne & Orr, 2004; Price, 2008). The two subspecies *abietinus* and *tristis* differ in plumage color, vocalizations, size, and habitat preference, and they have distinct breeding and wintering grounds except for a zone around the Ural mountains where they co‐occur during breeding season (del Hoyo et al., 2006; Komarova & Shipilina, 2010; Marova & Alekseev, 2008; Marova et al., 2009, 2013; Svensson, 1992). Given the specific characteristics of each subspecies and the relatively deep divergence (1.5–2% mtDNA divergence; Helbig et al., 1996), one can assume that both local adaptation and stochastic processes during allopatric separation have played a role in driving phenotypic divergence and that individuals with intermediate characters resulting from hybridization and backcrossing may have reduced fitness as a consequence of lower attractiveness to mates, intermediate migration patterns, and nonoptimal utilization of specific habitats (Rundle & Nosil, 2005). In support of that, allopatric *tristis* and *abietinus* show negligible reactions to the other subspecies' vocalizations (Marova et al., 2009; Marova et al. 2017; Martens & Meinche, 1989), indicating that song recognition could be a strong prezygotic barrier. However, within sympatric regions, chiffchaff of both subspecies respond more actively to alien song types—the underlying mechanism behind that difference is not known (Marova et al., 2009). In addition, both *abietinus* and *tristis* females have been observed pairing up with males with intermediate plumage characters in the field (unpublished observations) indicating that potential prezygotic barriers related to both vocalizations and morphology are still permeable. Our analysis of genomic composition of birds within the sympatric zone showed that several individuals express diagnostic plumage characters and perform diagnostic song despite harboring the foreign mtDNA type and/or a considerable proportion of foreign nuclear alleles which indicates that the genetic elements underlying morphological and vocalization differences occur in a restricted portion of the genome. It is too early to speculate if these regions also contain genes of importance for reproductive isolation. However, investigating the genome‐wide pattern of differentiation for a much larger set of nuclear markers and investigating the genomic admixture proportions in birds with intermediate phenotypes will give a chance to identify regions that show elevated differentiation and assess if those have appeared as a result of restricted gene flow in particular genomic regions or as a consequence of adaptation to different habitats during separation (Harrison & Larson, 2016; Wolf & Ellegren, 2016).

It is likely that the current region of sympatry is a secondary contact zone formed after recolonization from glacial refugia, and such zones might have formed recurrently during warmer periods during the Pleistocene glaciation cycles allowing for episodic interspecific gene flow. We found that introgression seem to be biased with a higher proportion of *tristis* mtDNA haplotypes and nuclear alleles within the sympatric region meaning that backcrossing predominantly occurs into the *tristis* lineage. This directional introgression may explain the presence of birds with somewhat yellowish plumage in the west Siberian plain (see discussion above on “fulvescens”), as these could carry a fraction of *abietinus* alleles that have spread through the *tristis* population eastwards by recurrent backcrossings. As our sampling was restricted to only include males, we are unfortunately not able to compare to the geographic distribution of mtDNA haplotypes and correlation between mtDNA and genomic composition in females. As many avian taxa have female‐biased dispersal (Mabry, Shelley, Davis, Blumstein, & Van Vuren, 2013) and a comparatively high rate of mtDNA introgression across divergent lineages (reviewed in Rheindt & Edwards, 2011), it is plausible that additional analyses including female samples from across the distribution ranges of *abietinus* and *tristis* would provide more quantitative information on the presence and potential bias in introgression patterns across the two subspecies.

The introgression bias into *tristis* appeared to be stronger in the northern sympatric region. It is known from previous analyses that the southern Urals probably served as a refugium with forest vegetation that preglacial fauna of Europe inhabited during glaciation (Vorontsov, 1949). It is possible that the ancestors of current European chiffchaff have been present in this region also during glacial maxima. After the retreat of the ice cover, Siberian chiffchaff then probably recolonized Siberia from the southeast from one (or both) of the Siberian Altai/Sayan refugia (Nazarenko, 1982, 1985) coming in contact with European chiffchaff in the southern Urals relatively early after the ice sheet retracted. The Arkhangelsk region, in contrast, was fully covered by glaciation, and both European and Siberian chiffchaff probably colonized that area much later indicating that the northern sympatric region is younger than the southern zone. If hybridization reduces fitness, as might be expected given the considerable differences in plumage, song, and migration routes, a longer history of secondary contact may have led to increased reinforcement of prezygotic barriers in the southern sympatric region, decreasing the rate of backcrossing into *tristis*, but it is not the only explanation for this pattern. Another aspect, albeit not mutually exclusive, relates to the abundance of each subspecies in different regions of the sympatric zone. In the northern zone, the predominant habitat type is boreal (coniferous) forest while the habitat is more fragmented in the southern zone with a patchy distribution of mixed and boreal forest. *Abietinus* is breeding in higher densities in mixed forest while *tristis* is more common in boreal (coniferous) forest and the relative density of *tristis* is higher in the northern zone (Komarova & Shipilina, 2010). This likely allows for more opportunities for backcrossing into *tristis* in the northern zone. Another question related to the biased introgression is whether *abietinus* and *tristis* differ in the ability to distinguish pure “consubspecifics” from hybrids/backcrosses. The observed pattern would then suggest that *tristis* are more prone to mate with hybrids but more data on mate preference will be needed before this can be elucidated.

In many avian study systems, there is clear evidence for male‐biased gene flow as a result of reduced fitness in female hybrids due to exposure of recessive incompatibility alleles on the Z‐chromosome (Haldane's rule, Haldane, 1922; Price, 2008). This has also been reported in another contact zone involving a different pair of chiffchaff species (Bensch, Helbig, Salomon, & Seibold, 2002). Our observation that one bird with *tristis* morphotype and >95% *tristis* nuclear SNPs actually harbored *abietinus* mtDNA indicates that female hybrids resulting from a cross between a male *tristis* and a female *abietinus* are fertile and can interbreed with *tristis* allowing for *abietinus* mitochondrial introgression into *tristis*. However, there is no individual in our data set that shows the opposite pattern (predominantly *abietinus* nuclear alleles and *tristis* mtDNA), all individuals with *tristis* mtDNA also have the largest proportion of diagnostic *tristis* nuclear SNPs. This could potentially be a result of low fitness of hybrid females from the reciprocal cross (male *abietinus*, female *tristis*), or that these hybrid females do not mate with *abietinus* males, but the sample size is limited, and extended sampling and mtDNA and nuclear genome analyses would be needed before this can be verified.

## 5 Conclusion

In this study, we combine genetic data with morphology and vocalizations across a hybrid zone between European (*abietinus*) and Siberian (*tristis*) chiffchaff. Our results indicate that the overall genetic differentiation between subspecies is low despite considerable phenotypic divergence. A large proportion of birds in the hybrid zone exhibit intermediate phenotypic characters and a mix of genetic ancestry, indicating extensive ongoing and past gene flow. Patterns of phenotypic and genetic variation vary between the northern and the southern part of the hybrid zone, probably reflecting differences in population densities and/or relative age of secondary contact. The data indicate that birds showing intermediate characters very likely represent individuals resulting from recurrent backcrossings and introgression of (predominantly) *abietinus* alleles into a *tristis* genomic background, and the previously described subspecies “*fulvescens”* is therefore not to be considered a distinct taxon. The data also point to the difficulties in identification of specific individuals based on phenotypic characters alone.

## Conflict of Interests

The authors declare no financial or nonfinancial conflict of interests.

## Supporting information


**Figure S1** Approximate distribution ranges of *P. c abietinus* (green) and *P. c. tristis* (yellow)
**Figure S2** An illustration of the different steps in the generation and analysis of nuclear DNA
