# Supplementary material for: Patterns of genetic, phenotypic, and acoustic variation across a chiffchaff (Phylloscopus collybita abietinus/tristis) hybrid zone
Source: Ecol Evol. 2017 Mar 2;7(7):2169–80. doi: 10.1002/ece3.2782 (PMC5383471; doi:10.1002/ece3.2782)

Supplementary Figure S1.

Approximate distribution ranges of *P. c abietinus* (green) and *P. c. tristis* (yellow). Numbers refer to sampling locations for all samples included in the study. Sample sizes for each respective location are listed in the table. * the same individual may be present in several categories of characteristics.

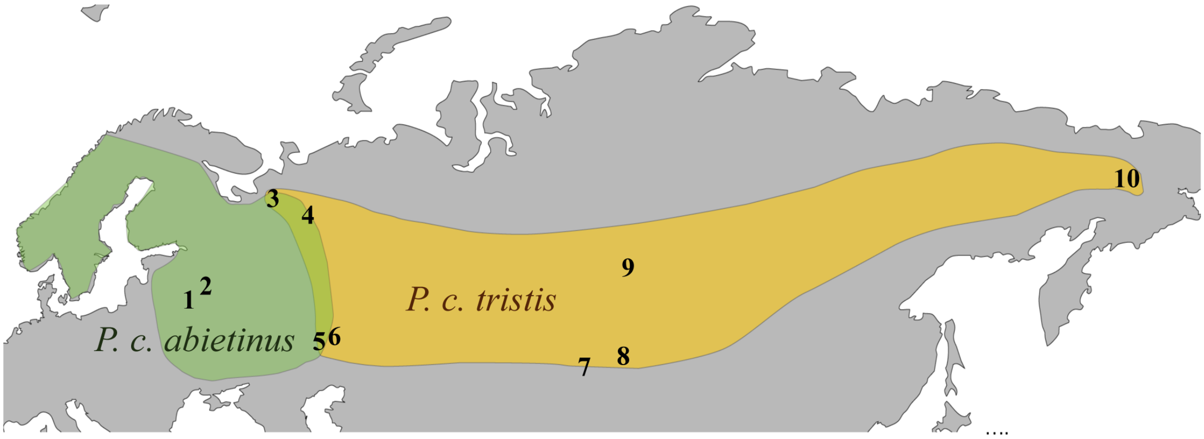

	Location	# of individuals *	Morphology (n=228)	Song (n=138)	mtDNA (n=192)	IDs of genotyped (NGS) birds
1	Moscow, Vladimir region	10	10	10	6	A6-A7
2	Kostroma region	6	6	6	5	A1-A5
3	Arkhangelsk region	52	51	21	48	N1-N10
4	Komi republic	15	12	10	5	-
5	Southern Ural National Reserve	105	93	44	75	S1-S10
6	Ilmenski National Reserve	20	20	11	16	-
7	Altai mountains	3	3	3	3	T9
8	Sayan mountains	11	11	11	11	T1-T4
9	Middle Enisey	22	22	22	22	T5-T8
10	Chukotka	1	1	-	1	T10

Supplementary Figure S2.

An illustration of the different steps in the generation and analysis of nuclear DNA.

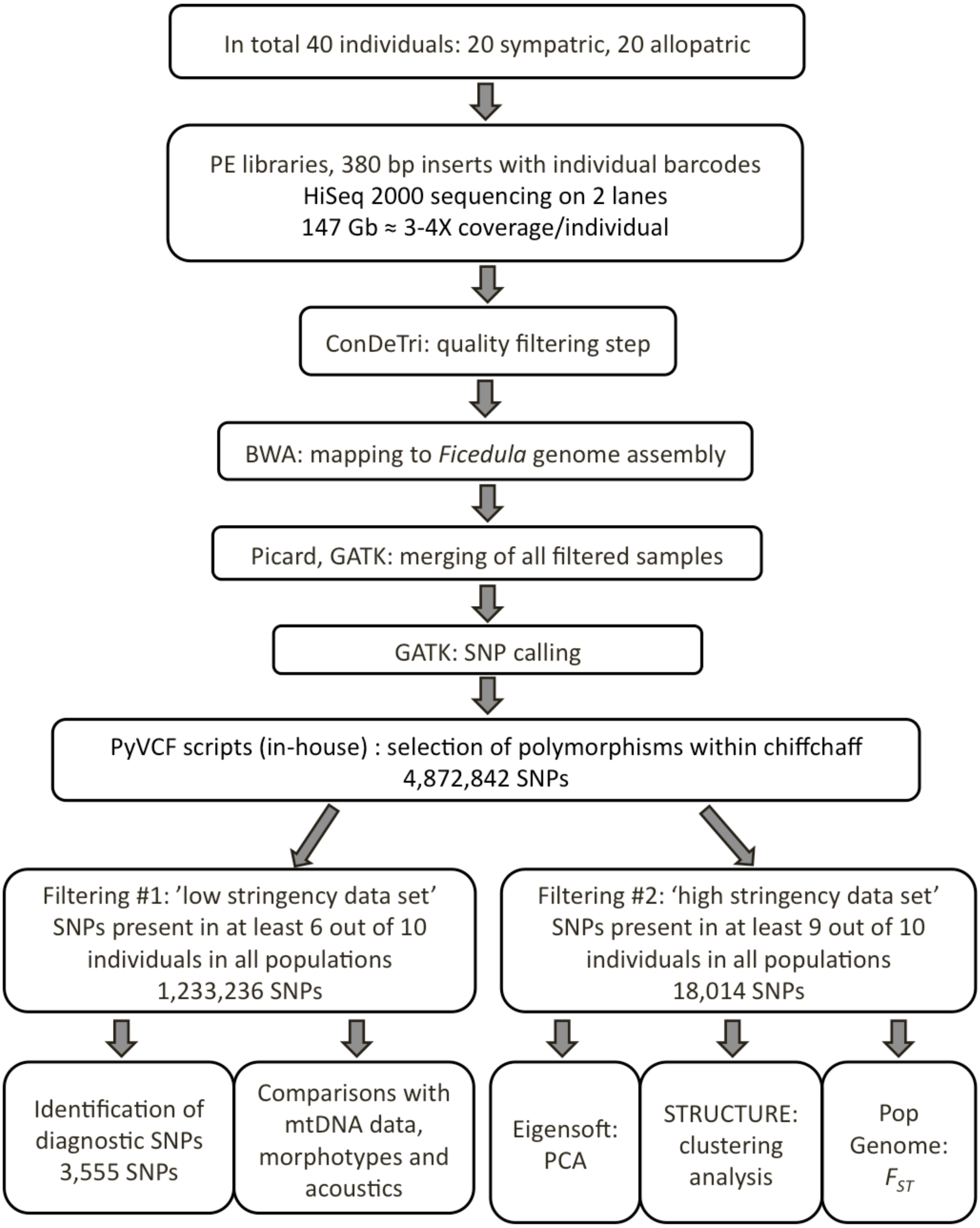

Supplement: Supplementary file 1 — Figure S1 Approximate distribution ranges of P. c abietinus (green) and P. c. tristis (yellow) Figure S2 An illustration of the different steps in the generation and analysis of nuclear DNA [file ECE3-7-2169-s001.pdf]
